# Factors associated with influenza vaccine adherence among health care workers in Abu Dhabi

**DOI:** 10.4102/sajid.v40i1.696

**Published:** 2025-07-15

**Authors:** Premilla Keerthy, Lizeth Roets

**Affiliations:** 1 Department of Health Studies, Faculty of Social Sciences, University of South Africa, Pretoria, South Africa

**Keywords:** influenza, vaccination, health care worker, Abu Dhabi, health belief model, vaccine adherence, UAE hospital

## Abstract

**Background:**

Influenza outbreaks rapidly threaten public health, making vaccination a critical tool in reducing transmission. Health care workers (HCWs), especially those in direct patient care roles within all health care settings, are encouraged to receive annual influenza vaccinations to enhance their immunity and ensure patient safety.

**Objectives:**

This study aimed to identify and describe the factors associated with HCWs’ adherence to annual influenza vaccination within a United Arab Emirates (UAE) hospital and provide recommendations to improve adherence rates.

**Method:**

Using a quantitative approach, a questionnaire based on the Health Belief Model and existing literature was administered to all 2,080 staff members of the UAE hospital under study. Of these, 1018 participants completed the questionnaire.

**Results:**

The study uncovered several key factors influencing HCWs’ influenza vaccine uptake. Notably, social influence from colleagues emerged as a significant factor, alongside barriers, such as inconvenient vaccination times, limited awareness of hospital policies, an absence of follow-up by the hospital administration, a lack of prior influenza cases and fear of injections. Motivational factors included the desire to protect friends and family, employer recommendations, mandatory policies and HCWs’ intrinsic motivation to safeguard patients.

**Conclusion:**

The findings of the study informed recommendations to enhance vaccination rates. These recommendations include health education materials, social media awareness campaigns, mandatory vaccination policies, on-duty vaccination services, free vaccination, flexible post-vaccination recovery time, special sick leave policies and vaccine benefit awareness.

**Contribution:**

These suggestions enable health care organisations to boost employee influenza vaccination rates, aiding public health efforts to cope with seasonal influenza outbreaks.

## Introduction

Most people agree that vaccination is the most important component of public health initiatives to reduce the spread of infectious illnesses. Many vaccinations are now being developed and marketed as a preventative measure against contagious diseases, particularly for high-risk populations.^1^ One prevalent disease that might cause pandemics if vaccination regimens are not implemented to control it, is the influenza virus infection.

Influenza outbreaks are dangerous to public health and they recur on a regular basis. Because the influenza virus attacks respiratory mucosa, it may cause severe sickness and high mortality rates (about 1.8 people per 100 000) because of complications, such as pneumonia.^2^ Young, aged and chronically sick people are more likely to die from influenza virus infections.^3^ However, because of modern medical advances, these patients are afforded the opportunity to obtain effective vaccination against annual influenza A (H1N1; H3N2) and influenza B outbreaks. Nursing staff have been linked to the spread of the virus.^4^ Health care workers (HCWs) may be infected with the virus from close contact with patients, which may then spread to other staff, as well as vulnerable patients.^5^ Research shows that, in general, there is at least a 5.5% relative risk that patients will catch influenza from infected HCWs.^6^ The risk of flu escalates in HCWs to 22.6% when in close contact with an infected patient, and a contagious health care professional.^7^ Health care workers, particularly those who provide patient care and handle human tissues and body fluids, should acquire flu shots to increase their immunity and improve patient safety.^8,9^

The case for promoting influenza vaccination of HCWs has been made because it reduces needless and preventable absences during a time of high demand for health care services.^10^ Reliable evidence is provided, by documented studies, which shows that many cases of HCW absenteeism, during influenza epidemics, are caused by a lack of preventive measures (such as immunisation).^11^ The coronavirus disease 2019 (COVID-19) pandemic, and the ensuing deficiency of resources to stop HCW transmission, are evidence of the significance of preventative interventions, including vaccinations, to guarantee HCW availability and enhance the quality of patient care.^12^

Research indicates that the past influenza vaccine’s effectiveness declines over time and that the chance of contracting the illness increases as time elapses after vaccination.^13^ Consequently, postponing vaccination could lead to fewer possibilities for immunisation. Influenza vaccinations have been shown to have an efficacy of between 70.5% and 90.5% in preventing infections among HCWs.^9^ Nonetheless, little research in the UAE concentrates on the influenza vaccine.^14^

It has been determined that several factors can affect people’s views of how deadly influenza is. Vaccination history and heightened sensitivity are examples.^15^ Health care personnel who have received the vaccination regard influenza as a greater health threat than those who have not.^16^ Health care personnel who have treated influenza patients, had higher influenza vaccination adherence rates.^10^ According to research,^17^ these health care personnel have greater vaccine adherence rates because they believe they are more susceptible to illness, have more cues to act and have fewer hurdles.

The main factors associated with influenza vaccination are HCW susceptibility, severity of influenza infections, benefits of vaccination, psychological factors, concerns about vaccine safety and efficacy, cues to action, demographic characteristics, ethical considerations, accessibility to the influenza vaccine, awareness of the importance of adherence, cultural factors and self-efficacy.^18^

This study evaluated the vaccination-related factors for vaccine adoption among Abu Dhabi’s HCWs.

## Methodology

### Research design

This cross-sectional study^19^ employed questionnaires to gather quantitative data on factors influencing HCWs adherence to influenza vaccination. The study site was a major 500-bed academic tertiary care teaching hospital in Al-Ain, chosen for its multi-cultural HCW population and accessibility. The accessible population included 1100 nurses, 536 doctors, 102 pharmacists and 342 clinical staff. From the total assessable population of 2080 HCWs, 1018 completed questionnaires through purposive sampling (48.9% response rate).

### Questionnaire development

The researcher developed a questionnaire with 13 open-ended and 22 closed-ended questions and after ethical approval from the custodian University as well as the hospital under study, undertook a pilot study with 42 respondents (6 from each HCW group such as Physicians, Nurses, Physiotherapists, Respiratory Therapists, Radiographers, Pharmacists and Radiotherapy Technicians). Participants (*n* = 42) in the pilot study did not take part in the main study. Risks were minimised by fully informing HCWs about the survey, ensuring confidentiality and allowing withdrawal without consent.

### Data collection

Department heads served as gatekeepers in the hospital, for recruiting volunteers, and informing HCWs about data-collection dates. Gatekeepers received an information letter outlining their responsibilities, which were coordinating questionnaire distribution and collection. Gatekeepers distributed information letters and self-administered questionnaires from the researcher to HCWs, who did provide written informed consent. Health care workers had 4 days to complete and submit questionnaires via drop boxes in their units, ensuring confidentiality.

The researcher and statistician analysed the quantitative data from closed-ended questions using Excel and SPSS v22. Descriptive statistics, frequencies, means and correlation analyses, explored variables influencing vaccine adherence. The researcher employed open coding on open-ended responses to extract themes, categories and sub-themes related to vaccine adherence.

### Ethical considerations

Ethical clearance to conduct this study was obtained from the University of South Africa Health Studies Higher Degrees Committee (No. REC-012714-039). Prior to data collection, written informed consent was obtained from all human participants. All participant data were de-identified and coded with unique identifiers to maintain confidentiality. Only the research team had access to the key linking the identifiers to the participants. All paper-based records were stored in a locked cabinet, and all electronic records were password-protected. Measures were taken to minimise risks to participants, such as allowing them to skip any questions that made them uncomfortable and informing them of their right to withdraw from the study at any time without consequences. Results were reported only in aggregate form to protect confidentiality further.

## Results

In total, 2080 questionnaires were distributed and only 1018 (48.9%) of the questionnaires were received back and were valid and suitable for further analysis after data cleaning. The sample size (*N* = 1018) was fit to generate findings with a robust statistical strength and high generalisability capability. All the data were checked for accuracy of the entered data outliers and other variances. All these activities were performed in an Excel spreadsheet and later exported to SPSS for analysis. To promote the means analysis of the four-point Likert scale, the following data ranges were used to analyse the computed means: ‘Strongly disagree’ was represented by the range of 1–1.74; ‘Disagree’ from 1.75 to 2.49; ‘Agree’ from 2.5 to 3.24 and finally, ‘Strongly agree’ was represented by the values between 3.25 and 4.0. For the open-ended qualitative question, 90 respondents provided comments, which were open-coded.

The findings revealed that 664 (65.2%) respondents were female, and 354 (34.8%) were male. Respondents younger than 25 (*n* = 18) and over 55 years (*n* = 115) represented only 1.8% and 11% of the sample, respectively. The HCWs between 26 and 35 years (*n* = 337) and those between 36 and 45 years (*n* = 317) represented 33.1% and 31.1% of the respondents, respectively. Finally, respondents between 46 and 54 years (*n* = 231) represented 22.7% of the sample. Respondents with health care experience of less than 1 year, between 1 and 2 years and between 3 and 4 years, represented 2% (*n* = 20), 3.3% (*n* = 34) and 5.9% (*n* = 60) of the respondents, respectively. Moreover, 10.5% (*n* = 107) of the respondents indicated they had between 5 and 7 years of experience. Most HCWs had worked in the health care industry for more than 7 years (*n* = 797, *f* = 78.3%). The sample consisted of 22.4% (*n* = 228) general physicians, 53.4% (*n* = 544) nurses, 9.2% (*n* = 94) radiographers, 4.7% (*n* = 48) physiotherapists, 4.5% (*n* = 46) pharmacists, 2.9% (*n* = 30) respiratory therapists, 2.1% (*n* = 21) radiotherapy technicians and 0.7% (*n* = 7) medical lab technologists and occupational therapists (‘others’). Physicians (*n* = 228, *F* = 214, *f* = 93.9%^1^), nurses (*n* = 544, *f* = 84.2%, *F* = 458), physiotherapists (*n* = 46, *F* = 40, *f* = 87%), respiratory therapists (*n* = 30, *f* = 86.7%, *F* = 26), radiographers (*n* = 94, *f* = 63.8%, *F* = 60), pharmacists (*n* = 46, *f* = 56.5%, *F* = 26) and radiotherapy technicians (*n* = 21, *f* = 90.5%, *F* = 19), respectively, had more than four hours of daily contact with patients. Approximately 5.9% (*n* = 32) of nurses spent less than an hour daily with patients, while 6.6% (*n* = 36) spent 1–2 h in direct contact with patients. Overall, 83.3% (*n* = 848) of the HCWs came into contact with patients for more than four hours per day, 2.8% (*n* = 28) HCWs had 3 to 4 h of daily contact, 4.7% (*n* = 48) HCWs had 1 to 2 h of daily contact and 9.2% (*n* = 94) of HCWs had daily contact of 1 h or less.

It was reported that 79% (*n* = 544, *F* = 430) of nurses, 96.5% (*n* = 228; *F* = 220) of physicians, 65.9% (*n* = 94; *F* = 62) of radiographers, 95.8% (*n* = 46; *F* = 46) of physiotherapists, 93.3% (*n* = 30; *F* = 28) of respiratory therapists, 47.8% (*n* = 46; *F* = 22) of pharmacists, 95.2% (*n* = 21; *F* = 20) of radiotherapy technicians and 100% (*n* = 7; *F* = 7) of medical lab technologists and occupational therapists (‘others’) adhered to the annual influenza vaccination. It was reported that 77.9% (*n* = 793, *N* = 1018) of the HCWs did not use other drugs to boost their immunity. Of the 225 HCWs who used other drugs, 210 indicated they mostly used multivitamins, followed by vitamin C, vitamin D, zinc, herbal medicines and B12 injections.

Several respondents offered comments in the open spaces provided in the questionnaire, which were open-coded. Themes, underpinned by categories, were identified, and direct quotations were used to illustrate how the categories were formed. The identified themes were perceived susceptibility to influenza, benefits to immunisation and perceived threats (barriers), as the health belief model suggested.

### Perceived benefits

Health-seeking behaviour is the perceived benefit of immunisation; thus, in this study’s context, the motivation to be vaccinated. The perceived benefits identified were self-protection, protecting friends and family and protecting patients, as identified in Table 1.

**TABLE 1:** Benefits to immunisation.

Perceived benefits	Job role	*N*	*F*	*F* (%)
Self-protection	Other (Medical Lab Technologists and Occupational Therapists)	7	5	71.40
	Physician	228	218	95.60
	Nurse	544	394	72.40
	Physiotherapist	48	42	87.50
	Respiratory Therapist	30	24	80.00
	Radiographer	94	62	66.00
	Pharmacist	46	22	47.80
	Radiotherapy Technician	21	20	95.20
Protecting friends and family from flu	Other (Medical Lab Technologists and Occupational Therapists)	7	5	71.40
	Physician	228	216	94.70
	Nurse	544	316	58.10
	Physiotherapist	48	32	66.70
	Respiratory Therapist	30	22	73.30
	Radiographer	94	48	51.10
	Pharmacist	46	18	39.10
	Radiotherapy Technician	21	20	95.20
Protecting patients	Other (Medical Lab Technologists and Occupational Therapists)	7	5	71.40
	Physician	228	218	95.60
	Nurse	544	324	59.60
	Physiotherapist	48	30	62.50
	Respiratory Therapist	30	16	53.30
	Radiographer	94	46	48.90
	Pharmacist	46	18	39.10
	Radiotherapy Technician	21	20	95.20

### Self-protection

In this study’s context, 71.4% (*n* = 7; *F* = 5) of the medical lab technologists and occupational therapists, 95.6% (*n* = 228; *F* = 218) of physicians, 72.4% (*n* = 544; *F* = 394) of nurses, 87.5% (*n* = 48; *F* = 42) of physiotherapists, 80% (*n* = 30; *F* = 24) of respiratory therapists, 66% (*n* = 94; *F* = 62) of radiographers, 47.8% (*n* = 46; *F* = 22) of pharmacists and 95.2% (*n* = 21; *F* = 20) of radiotherapy technicians, were motivated to get vaccinated to protect themselves from the flu.

### Protecting friends and family from influenza

It was indicated that 71.4% (*n* = 7; *F* = 5) of medical lab technologists and occupational therapists, 94.7% (*n* = 228; *F* = 216) of physicians, 58.1% (*n* = 544; *F* = 316) of nurses, 66.7% (*n* = 48; *F* = 32) of physiotherapists, 73.3% (*n* = 30; *F* = 22) of respiratory therapists, 51.1% (*n* = 94; *F* = 48) of radiographers, 39.1% (*n* = 46; *F* = 18) of pharmacists and 95.2% (*n* = 21; *F* = 20) of radiotherapists, stated that they were motivated to take the vaccine because they need to protect their friends and family from the flu.

### Protecting patients

The findings of the study were as 71.4% (*n* = 7; *F* = 5) of medical lab technologists and occupational therapists, 95.6% (*n* = 228; *F* = 218) of physicians, 59.6% (*n* = 544; *F* = 324) of nurses, 62.5% (*n* = 48; *F* = 30) of physiotherapists, 53.3% (*n* = 30; *F* = 16) of respiratory therapists, 48.9% (*n* = 94; *F* = 46) of radiographers, 39.1% (*n* = 46; *F* = 18) of pharmacists and 95.2% (*n* = 21; *F* = 20) of radiotherapists, stated they were motivated to be vaccinated because they wanted to protect their patients.

### Barriers to acceptance of vaccination

#### Convenient time for taking the vaccine

Only 3.5% (*n* = 228; *F* = 8) of physicians, 27.9% (*n* = 544; *F* = 152) of nurses, 25% (*n* = 48; *F* = 12) of physiotherapists, 20% (*n* = 30; *F* = 6) of respiratory therapists, 36.1% (*n* = 94; *F* = 34) of radiographers, 17.3% (*n* = 46; *F* = 8) of pharmacists, 19% (*n* = 21; *F* = 4) of radiotherapy technicians and 57.1% (*n* = 7; *F* = 4) of ‘others’, faced the challenge of a lack of convenient time to receive the vaccine, as noted in Table 2.

**TABLE 2:** Convenient time as barrier to acceptance.

Barriers to acceptance of vaccination	Job role	*N*	*F*	%
Convenient time for taking vaccines	Physicians	228	8	3.51
	Nurses	544	152	27.94
	Physiotherapists	48	12	25.00
	Respiratory Therapists	30	6	20.00
	Radiographers	94	8	17.39
	Pharmacists	46	4	19.05
	Radiotherapy Technicians	21	4	19.05
	Others (Medical Lab Technologists & Occupational Therapists)	7	4	57.41

#### Perceived ineffectiveness of the vaccine

In this study, some respondents also did not believe in the effectiveness of the vaccine.

#### Personal choice

Several respondents provided open-coded responses and mentioned personal reasons for not wanting to be vaccinated without any explanation.

#### Perceived threats: Previous cases of influenza-infected health care workers

The study’s findings revealed (see Table 3) that 4.3% (*n* = 228; *F* = 10) of physicians, 23.1% (*n* = 544; *F* = 126) of nurses, 25% (*n* = 48; *F* = 12) of physiotherapists, 33.3% (*n* = 30; *F* = 10) of respiratory therapists, 19.1% (*n* = 94; *F* = 18) of radiographers, as well as 100% (*n* = 7; *F* = 7) of ‘others’, were aware that there had been previous cases of infections among HCWs in the hospital. However, none of the pharmacists and radiography technicians reported previous personal infections, as noted in Table 3.

**TABLE 3:** Perceived threats.

Perceived threats	Job role	Agree		
		*n*	*F*	%
No previous cases of influenza-infected HCWs	Physician	228	10	4.39
	Nurse	544	126	23.16
	Physiotherapist	48	12	25.00
	Respiratory Therapist	30	10	33.33
	Radiographer	94	18	19.15
	Pharmacist	46	0	0.00
	Radiotherapy Technician	21	0	0.00
	Other	7	3	42.86
Fear of injection	Physician	228	15	6.58
	Nurse	544	136	25.00
	Physiotherapist	48	16	33.33
	Respiratory Therapist	30	12	40.00
	Radiographer	94	16	17.02
	Pharmacist	46	12	26.09
	Radiotherapy Technician	21	6	28.57
	Other	7	3	42.86

#### Fear of injection

In this study, 6.5% (*n* = 228; *F* = 15) of physicians, 25% (*n* = 544; *F* = 136) of nurses, 33.3% (*n* = 48; *F* = 12) of physiotherapists, 40% (*n* = 30; *F* = 10) of respiratory therapists, 17% (*n* = 94; *F* = 18) of radiographers, 26% (*n* = 46; *F* = 12) of pharmacists, 28.5% (*n* = 21; *F* = 6) of radiography technicians and 42.8% (*n* = 7; *F* = 3) of ‘others’, indicated their fear of injections prevented them from getting vaccinated, as noted in Table 3.

#### Scared of the effects

Respondents indicated they feared the adverse effects of influenza vaccinations. When the HCWs were asked about their awareness of the side effects and whether or not they were informed of the side effects by their doctors, 35.9% (*n* = 365, *N* = 1018) strongly agreed, 51% (*n* = 519, *N* = 1018) agreed, 12.8% (*n* = 130, *N* = 1018) disagreed, and the remaining 0.4% (*n* = 4, *N* = 1018) strongly disagreed.

#### Allergies and health issues

Some respondents stated that they have allergies, which will likely create complications with the influenza vaccination and, therefore, they will not take the risk.

#### Cues to action: Social influence from colleagues

It was reported that 6.1% (*n* = 228; *F* = 14) of physicians, 20.9% (*n* = 544; *F* = 114) of nurses, 20.8% (*n* = 48; *F* = 10) of physiotherapists, 13.3% (*n* = 30; *F* = 4) of respiratory therapists, 34.0% (*n* = 94; *F* = 32) of radiographers, 43.4% (*n* = 46; *F* = 20) of pharmacists, 9.5% (*n* = 21; *F* = 2) of radiotherapy technicians and 57.1% (*n* = 7; *F* = 4) of ‘others’, indicated that their colleagues socially influence them through peer pressure, as noted in Table 4.

**TABLE 4:** Cues to action.

Cues to Action	Job role	*N*	*F*	%
Social influence from colleagues	Physician	228	14	6.14
	Nurse	544	114	20.96
	Physiotherapist	48	10	20.83
	Respiratory Therapist	30	4	13.33
	Radiographer	94	32	34.04
	Pharmacist	46	20	43.48
	Radiotherapy Technician	21	2	9.52
	Other	7	4	57.14
The employer recommends the vaccine	Other (Medical Lab Technologists and Occupational Therapists)	7	3	42.90
	Physician	228	210	92.10
	Nurse	544	296	54.40
	Physiotherapist	48	22	75.00
	Respiratory Therapist	30	26	86.70
	Radiographer	94	40	42.60
	Pharmacist	46	16	34.80
	Radiotherapy Technician	21	18	85.70
Lack of follow-up by the hospital administration	Physician	228	8	3.51
	Nurse	544	84	15.44
	Physiotherapist	48	6	12.50
	Respiratory Therapist	30	6	20.00
	Radiographer	94	20	21.28
	Pharmacist	46	4	8.70
	Radiotherapy Technician	21	2	9.52
	Other	7	7	100.00
Employers enforced vaccination	Other (Medical Lab Technologists and Occupational Therapists)	7	3	42.90
	Physician	228	204	89.50
	Nurse	544	260	47.80
	Physiotherapist	48	22	45.80
	Respiratory Therapist	30	14	46.70
	Radiographer	94	36	38.30
	Pharmacist	46	10	21.70
	Radiotherapy Technician	21	18	85.70

#### The employer recommends the vaccine

It was reported that 42.9% (*n* = 7; *F* = 3) of medical lab technologists and occupational therapists, 92.1% (*n* = 228; *F* = 210) of physicians, 54.4% (*n* = 544; *F* = 296) of nurses, 75% (*n* = 48; *F* = 22) of physiotherapists, 86.7% (*n* = 30; *F* = 26) of respiratory therapists, 42.6% (*n* = 94; *F* = 40) of radiographers, 34.8% (*n* = 46; *F* = 16) of pharmacists and 85.7% (*n* = 21; *F* = 18) of radiotherapy technicians, stated they feel motivated to take the influenza vaccine because their employer recommends it.

#### Lack of follow-up by the hospital administration

In this study, 3.5% (*F* = 8; *n* = 228) of physicians, 15.4% (*n* = 544; *F* = 84) of nurses, 12.5% (*n* = 48; *F* = 6) of physiotherapists, 20% (*n* = 30; *F* = 6) of respiratory therapists, 21.8% (*n* = 94; *F* = 20) of radiographers, 8.7% (*n* = 46; *F* = 4) of pharmacists, 9.5% (*n* = 21; *F* = 2) of radiography technicians and 100% (*n* = 7; *F* = 7) of ‘others’ perceived a lack of follow-up by the hospital’s administration as a barrier to their influenza vaccination.

#### Employers enforced vaccination

It was indicated that 42.9% (*n* = 7; *F* = 3) of ‘others’ (medical lab technologists & occupational therapists), 89.5% (*n* = 228; *F* = 204) of physicians, 47.8% (*n* = 544; *F* = 260) of nurses, 45.8% (*n* = 48; *F* = 22) of physiotherapists, 46.7% (*n* = 30; *F* = 14) of respiratory therapists, 38.3% (*n* =9 4; *F* = 36) of radiographers, 21.7% (*n* = 46; *F* = 10) of pharmacists and 85.7% (*n* = 21; *F* = 18) of radiotherapists, stated that their employers forced them to get vaccinated.

#### Self-efficacy

Knowledge about the hospital’s policy regarding influenza uptake: It was reported that 16.2% (*n* = 228; *F* = 37) of physicians, 86.7% (*n* = 544; *F* = 472) of nurses, 100% (*n* = 48; *F* = 48) of physiotherapists, 80% (*n* = 30; *F* = 24) of respiratory therapists, 82.9% (*n* = 94; *F* = 78) of radiographers, 86.9% (*n* = 46; *F* = 40) of pharmacists, 100% (*n* = 21; *F* = 21) of radiography technicians and 100% (*n* = 7; *F* = 7) of ‘others’, agreed that they knew the hospital’s policy regarding influenza uptake. In addition, 82% (*n* = 804, *N* = 982) of the HCWs noted that the hospital has an influenza policy, while 17% (*n* = 170, *N* = 982) were unsure whether there is an influenza policy. The remaining 1% (*n* = 8, *N* = 982) of HCWs stated that their hospital had no influenza policy, as noted in Table 5.

**TABLE 5:** Self-efficacy.

Self-efficacy	Job role	*N*	*F*	%
Knowledge about the hospital’s policy regarding influenza uptake	Physician	228	37	16.23
	Nurse	544	472	86.76
	Physiotherapist	48	48	100.00
	Respiratory Therapist	30	24	80.00
	Radiographer	94	78	82.98
	Pharmacist	46	40	86.96
	Radiotherapy Technician	21	21	100.00
	Other	7	7	100.00

## Discussion

Demographics included gender, age group, health care experience, work length and professional position. The female and male ratio for all HCWs (65.2%:34.8%) aligns with global studies indicating low male nursing proportions, projected as 79% female nurses in the Middle East by 2022.^20,21^ Findings corroborate research^22^ observing low vaccine uptake among nurses, radiologists and pharmacists. Despite high physician uptake, 4.3% did not think vaccines were required, which is similar to complementary or alternative medicine practitioners with poor adherence.^23^ Hospitals with voluntary programmes reported low adherence.^24^ However, an uptake of 82% was achieved here despite it being voluntary, likely because of the active promotion of vaccination for all HCWs.

Compared to the US, which reported vaccination rates of 77.3% among HCWs, the UAE has demonstrated a higher rate of influenza immunisation (82%).^25^ The results contradict research,^26,27^ which reported that coverage rates had dropped below 50% in areas where influenza epidemics have not occurred recently. Although there have never been influenza epidemics in the UAE, this study found an 82% vaccination rate, which is significantly higher than the cutoff rate, which is the minimum required (below 50%).

The hospital that was the subject of the study, had an 82% vaccine rate, which was below the recommended level of 90% in health care facilities, according to CDC recommendations. Therefore, to cross the suggested barrier, an additional 8% margin had to be reached.^28^

The Health Belief Model (HBM) was appropriate for addressing HCWs’ vaccination behaviour, so data were interpreted using this model. The theoretical framework guided the presentation of the findings based on the model’s constructs. Both closed and open-ended questions identified barriers to vaccination adherence, examining the factors that are potentially affecting health-seeking behaviour and difficulties for HCWs. Perceived vulnerability, an important HBM component, was assessed through open-ended questions. Health care workers were hesitant to take the patient role.^29^ Most did not perceive themselves as an at-risk population requiring preventive actions,^30^ as people will not act unless they believe there is a significant health risk.^31^

The HBM states that perceived benefits influence health-seeking behaviour; vaccination motivation determines this study. Three identified perceived benefits were protecting oneself, friends, family and patients – pointing to causes and incentives driving annual influenza vaccination among HCWs. Self-protection against the flu is a major driver.^32^ Participants were motivated by self-protection, with open-ended responses indicating the main advantage was self-protection. Self-protection motivated uptake and adherence.^33^ Reported benefits aligned with the literature that people adopt healthy behaviours if they reduce perceived illness risk.^18^ Health care workers were encouraged to get vaccinated to protect themselves, their families and their friends from the flu.^11^ Self-defence motivated all HCWs except physicians (95.6% motivated by these factors). Pharmacists had the lowest motivation (47.8%) from self-defence.

Research^33^ states that the perceived benefits of vaccination for HCWs include reducing transmission to friends/family and encouraging uptake. Most participants were motivated to protect friends and family from the flu. Common factors, like the risk of transmitting flu to loved ones, can encourage HCW vaccination.^34^ Motivation to protect friends and family was present in 95.2% of radiation technicians, 94.7% of physicians, 73.3% of respiratory therapists, 71.4% of ‘others’ and 58.1% of nurses – contrasting the claim^34^ that few nurses consider community health for flu vaccination. Over half of the nurses were driven by protecting friends/family, demonstrating the regional uniqueness of UAE, where over two-thirds of HCWs view community health as a driver for adherence.

Some HCWs are motivated by protecting patients, rather than feeling compelled.^35,36^ However, only 59.6% of nurses who directly interact with patients said they would vaccinate to protect patients.^37^ Many are encouraged, not mandated, to vaccinate because of caring duties and not wanting to harm patients, with the Hippocratic Oath driving adherence.^35,36,38^

One issue influencing vaccination adherence is timing.^35,39,40^ Mobile carts providing on-the-spot immunisations were suggested to increase adherence by allowing HCWs to follow schedules.^41,42^

Many HCWs do not believe vaccines will protect them against influenza^43,44,45^ or COVID-19.^46^ Some participants doubted the vaccine’s efficacy and provided vague or no reasons for not vaccinating.^47^ Health care workers felt that vaccination rates declined when there were no reported influenza cases, as they perceived a diminished danger.^35^ However, fear of injections,^48,49^ concerns about effectiveness and safety,^50,51^ and weighing benefits against risks,^52^ also influenced decisions. Some feared allergic reactions and perceived health costs.^35^

Social influence from co-workers impacted adherence, aligning with findings that a lack of supportive colleagues was a barrier.^33^ Health care workers were more likely to adhere when occupational health units recommended vaccines, similar to patterns for other vaccines.^53,54^ Some nurses were prepared to support adherence if recommended by the occupational health unit.^35^ Participants felt that the lack of hospital follow-up hindered their flu vaccination, corroborating previous findings that the lack of cue to action in hospitals impedes HCW immunisation rates. However, not all participants saw the lack of follow-up as a barrier, as cues like billboards and notifications did not affect HCWs’ view of flu as a threat.^55^

Mandating flu vaccination can inspire and boost HCW adherence rates.^11^ However, mandatory COVID-19 laws sparked protests and strikes in some countries like the US.^56^ Respondents had mixed views on compulsory vaccination – only 89.5% of physicians said mandates increased flu shot uptake, possibly because of other HCWs valuing freedom of choice.^57^ Health care workers complied as employers mandated vaccination. So, while making it mandatory is suitable for increasing adherence,^11^ it also faced opposition to COVID-19 mandates.^56^

Knowledge influences health-seeking behaviour and vaccine uptake.^58,59^ This study supports research^60^ that found low adherence rates can be caused by several factors, including not knowing the yearly influenza vaccine requirements. Thus, ignorance reduced vaccination rates.^60^ Promotional (emails and flyers) and instructional (meetings and campaigns) efforts seem to be working well at the hospital.^53^ Even with these calls to action, just 82% of health care professionals receive the necessary yearly vaccines.

## Conclusion

This study on factors associated with influenza vaccine adherence among HCWs found that demographic factors like gender, age, experience and position, influenced vaccination rates, with lower uptake among nurses, radiologists and pharmacists than physicians. While the hospital where the study was conducted had a relatively high (82%) HCW vaccination rate, it fell short of the CDC’s 90% recommendation for health care facilities. The Health Belief Model helped interpret findings, highlighting perceived susceptibility, benefits, threats, and cues to action, as drivers of vaccination. Motivators included self-protection and protecting family, friends, and patients, while barriers included concerns over effectiveness, side effects and access. To improve adherence, future efforts should address vaccine hesitancy, increase convenient access, provide education on benefits and safety and tailor interventions to different HCW groups’ needs. This can enhance influenza prevention in health care settings by achieving higher vaccination among this critical workforce. Finally, although the study was conducted in the UAE, the same factors may be relevant to HCWs in different contexts. Consequently, the recommendations could be applicable to a broader audience.

## Footnotes

1 *F* stands for frequency within *n* and *f* % stands for percentage frequency within *n*.
